# Proposed Mechanism-Based Risk Stratification and Algorithm to Prevent Sudden Death in Epilepsy

**DOI:** 10.3389/fneur.2020.618859

**Published:** 2021-01-25

**Authors:** Michael Lucchesi, Joshua B. Silverman, Krishnamurthi Sundaram, Richard Kollmar, Mark Stewart

**Affiliations:** ^1^Department of Emergency Medicine, State University of New York Health Sciences University, Brooklyn, NY, United States; ^2^Department of Otolaryngology, North Shore Long Island Jewish Medical Center, New Hyde Park, NY, United States; ^3^Department of Otolaryngology, State University of New York Health Sciences University, Brooklyn, NY, United States; ^4^Department of Cell Biology, State University of New York Health Sciences University, Brooklyn, NY, United States; ^5^Department of Neurology, State University of New York Health Sciences University, Brooklyn, NY, United States; ^6^Department of Physiology & Pharmacology, State University of New York Health Sciences University, Brooklyn, NY, United States

**Keywords:** apnea, laryngospasm, SUDEP, airway obstruction, respiratory arrest

## Abstract

Sudden Unexpected Death in Epilepsy (SUDEP) is the leading cause of death in young adults with uncontrolled seizures. First aid guidance to prevent SUDEP, though, has not been previously published because the rarity of monitored cases has made the underlying mechanism difficult to define. This starkly contrasts with the first aid guidelines for sudden cardiac arrest that have been developed based on retrospective studies and expert consensus and the discussion of resuscitation challenges in various American Heart Association certificate courses. However, an increasing amount of evidence from documented SUDEP cases and near misses and from animal models points to a consistent sequence of events that starts with sudden airway occlusion and suggests a mechanistic basis for enhancing seizure first aid. In monitored cases, this sudden airway occlusion associated with seizure activity can be accurately inferred from inductance plethysmography or (depending on recording bandwidth) from electromyographic (EMG) bursts that are associated with inspiratory attempts appearing on the electroencephalogram (EEG) or the electrocardiogram (ECG). In an emergency setting or outside a hospital, seizure first aid can be improved by (1) keeping a lookout for sudden changes in airway status during a seizure, (2) distinguishing thoracic and abdominal movements during attempts to inspire from effective breathing, (3) applying a simple maneuver, the laryngospasm notch maneuver, that may help with airway management when aggressive airway management is unavailable, (4) providing oxygen early as a preventative step to reduce the risk of death, and (5) performing cardiopulmonary resuscitation before the limited post-ictal window of opportunity closes. We propose that these additions to first aid protocols can limit progression of any potential SUDEP case and prevent death. Risk stratification can be improved by recognition of airway occlusion, attendant hypoxia, and need for resuscitation.

## Introduction

Sudden unexpected death in epilepsy (SUDEP) is a major cause of death among children and adults with epilepsy, particularly individuals whose seizures are poorly controlled [e.g., (1, 2)]. The prevalence of epilepsy in the US population is ~1% and between 2 and 17% of deaths in these patients are labeled SUDEP [e.g., (3)], the single most common cause of death among persons with epilepsy (4, 5). Mortality rates in adults with epilepsy are 2–3 times greater than their non-epileptic counterparts (6, 7). Fortunately, the incidence of sudden death in epilepsy is low (8–11), but it is the rarity of the condition that contributes to its unexpected presentation and paucity of detailed physiological data. Given the elusive mechanism of death and a diffuse risk profile that includes: (1) highest SUDEP risk when seizure control is poor, (2) unpredictable, often nocturnal timing of life-threatening or fatal events, and (3) an apparent absence of significant pre-existing cardiovascular pathology, SUDEP prevention has centered on prevention of seizures [e.g., (12)].

The Mortality in Epilepsy Monitoring Unit Study (MORTEMUS) identified a consistent sequence of events in epilepsy patients beginning with a generalized tonic clonic seizure and ending in death (10). Curated cases with detailed records from multiple epilepsy centers were used to demonstrate a sequence of events from the end of the seizure consisting of “terminal” apnea, bradycardia, and asystole. Other work in rats and mice showed that seizure-associated laryngospasm could cause obstructive apnea that may persist to the point of respiratory arrest (13–15). The linkage between preclinical and clinical data was formed by EMG evidence of inspiratory attempts and specific changes in cardiac rhythm (14, 16, 17) [see also Supplemental Data in Ryvlin et al. (10)].

The goal of this review is to make first-responders look out for abrupt changes in airway status during a seizure and to offer preventative as well as interventional steps against the life-threatening consequences of obstructive apnea. First, we want to emphasize that a dangerous mechanism for the airway status to abruptly change from open to closed is internal, i.e., laryngospasm, making airway obstruction unexpected and thus potentially catching responders off guard. Second, we want to highlight the value of oxygen as a step that, when available early, protects against the consequences of obstructive apnea without actually influencing whether obstructive apnea occurs or not. Finally, we hope to raise awareness of the ictal and postictal sequence of events and time points for intervention in relation to the underlying mechanism so that efficacy of intervention steps can be optimized.

## Basic First Aid

Seizure first aid generally takes a “wait for the seizure to end” approach, emphasizing “care and comfort” first aid while remaining vigilant for uncommon severe features that would warrant a call for emergency help (18–21). According to one of the most detailed sets of guidelines (21), “During a convulsive or tonic-clonic seizure, it may look like the person has stopped breathing. As this part of a seizure ends, the muscles will relax and breathing will resume normally. Rescue breathing or CPR is generally not needed during these seizure-induced changes in a person's breathing.”

All seizure first aid protocols emphasize the importance of checking the airway and airway protection is emphasized in the “turn the person onto their side” guidance (https://www.epilepsy.com/learn/seizure-first-aid-and-safety/first-aid-seizures-stay-safe-side). First aid flowcharts for emergency departments are clear that cardiorespiratory compromise warrants oxygen as part of an active treatment (e.g., https://www.rch.org.au/clinicalguide/guideline_index/Afebrile_seizures/).

Critically, Ryvlin et al. (10) report on the successful resuscitation of potential SUDEP cases with cardiopulmonary resuscitation (CPR) applied within a narrow timeframe around the end of the seizure and terminal apnea. In the Ryvlin et al. dataset, 7/7 patients receiving CPR within 3 min of respiratory arrest (i.e., after “terminal apnea” occurred) were successfully resuscitated and 11/11 patients in whom CPR was initiated only after 10 min following respiratory arrest all died (10). It is the particular, narrow timeframe for CPR, and the reasons CPR may be necessary, that deserve discussion.

## Pathophysiology and Risk Stratification

Seizure-associated changes in cardiac rate and rhythm and respiratory rate and rhythm indicate seizure spread to medullary sympathetic premotor and parasympathetic motor neurons [e.g., (22, 23)] and adjacent respiratory brainstem regions [e.g., (24–27)]. Multiple pathways exist for the spread of seizure activity from subiculum to paraventricular nucleus (PVN) of the hypothalamus (28) and then from PVN to medullary regions [e.g., (29)], where seizure activity can access medullary autonomic nuclei, respiratory centers, and laryngeal motor neurons.

Respiratory derangements during seizures can be serious [reviewed in (2, 24, 30)]. Reports of ictal tachypnea, bradypnea, and apnea [e.g., (10, 13, 31–38)] all point to an impact of seizure activity on respiratory rhythm generation and thereby a role in oxygen desaturation during seizures (32, 36).

The significance of seizure-associated hypoxemia as a contributor to death has been shown in a number of animal models, including rats (13, 23, 39), mice (25, 40–42), cats (43, 44), and sheep (45, 46). In rats, seizure-associated obstructive apneic episodes were demonstrated with nerve recordings and laryngoscopy to be due to seizure spread to the recurrent laryngeal nerve (the principal motor nerve of the larynx), which caused severe laryngospasm and complete airway closure (13). Significant ST segment elevation indicative of hypoxia was also present in ECG recordings. Seizure-associated central apneic episodes, even when tens of seconds in duration, however, were associated with an open airway as observed with continuous laryngoscopy, modest decreases in heart rate, and no evidence of hypoxia in ECG records (13, 27). Importantly, Rheims et al. (47) used data from the REPO_2_MSE study to evaluate the occurrence and degree of post-ictal hypoxemia and found that early administration of oxygen was associated with an early recovery of oxygen saturation and less frequent post-ictal EEG suppression. While they leave the issue of central vs. obstructive apnea as “an open question,” they do demonstrate value for early oxygen with completely independent metrics.

An important step in defining the mechanism of SUDEP came from differentiation of first-order, seizure-induced pathophysiological events from second-order consequences of these first-order derangements (17). This review develops the arguments in favor of obstructive apnea and discusses potential alternative mechanisms for SUDEP in greater detail. Obstructive apnea, which occurs in a subset of seizures that include seizure-induced laryngospasm, was identified as the major cause of desaturation and death, but neither obstructive apnea nor laryngospasm has been specifically addressed in first aid guidance.

Laryngospasm has been suspected during seizures in patients or observed postictally, based on findings of stridor and a narrowed airway when attempting to place an endotracheal tube (48) or intensive inspiratory effort (16, 49), but ictal laryngospasm is difficult to directly assess in patients or animals [see e.g., (50–52)]. Pulmonary edema, a common finding at autopsy in SUDEP cases, is also indirect evidence of laryngospasm (53–55). Once obstructed, attempts to inspire against the closed glottis contribute to a rapid desaturation (13, 56). The hypoxemia and decreased cardiac output cause the seizure to abort, but the laryngospasm can persist to the point of respiratory arrest (defined as a cessation of attempts to inspire), followed ultimately by cardiac arrest (13, 14). Based on the clinical and animal data, respiratory arrest occurs very close in time to the end of seizure activity or the end of a motor convulsion [reviewed in (17)]. This work included a demonstration that early oxygen could significantly delay the time to respiratory arrest, even when available for a relatively short period of time before the onset of obstructive apnea (56). In mouse studies, early oxygen guaranteed survival in animals that had an extremely high probability of mortality (40, 41).

In persons who are monitored, seizure-associated airway occlusion can be accurately inferred from inductance plethysmography or (depending on recording bandwidth) electromyographic (EMG) bursts associated with inspiratory attempts appearing on the electroencephalogram (EEG) or the electrocardiogram (ECG). Evidence of significant hypoxia can appear as ST changes in the ECG or as EEG evidence of hypoxic seizure termination (decreasing amplitude with increasing frequency). With such data, individuals can be placed in a high-risk stratum that can be divided into three substrata when a seizure occurs with (1) evidence of airway occlusion only, (2) evidence of airway occlusion and significant hypoxia, or (3) requiring resuscitation.

For clarification, some additional discussion of central vs. obstructive apnea and the issue of stertor as a source of EMG is warranted. Concepts such as post-convulsive central apnea (PCCA) (57) have been put forth with mechanistic implications in SUDEP. For example, a paper by Vilella et al. (58) offered PCCA as a clinical biomarker for SUDEP. Their conclusions derive from a study of 148 seizures in 87 patients, none of whom died while being monitored, and only 21 seizures included airflow monitoring to distinguish central from obstructive apnea (59). Two patients were labeled as near misses (neither of which had airflow data) and a third patient died nearly 2 years after being recorded for the dataset. Twenty other patients displayed PCCA with no SUDEP concerns accounting for the lack of a statistical association of PCCA with SUDEP. This paper attempts to dismiss both laryngospasm and EMG signals associated with respiratory effort, in spite of the fact that the study data come from as early as 2011 when laryngospasm and obstructive apnea were not on most clinicians' radar.

With regard to the EMG signal associated with respiratory effort, Vilella et al. (58) imply that stertorous breathing is the explanation for respiration-related EMG signals appearing on EEG or ECG channels. Data on stertor in temporal relation to the EMG signals are not available and their own figures show the EMG signal in association with larger excursions on the inductance plethysmograms. As we reviewed in Stewart et al. (17), such respiratory effort-associated EMG signals were seen in (1) the MORTEMUS results (where stertorous breathing was either missed or not detected) (10), (2) in multiple animal experiments where the airway is known to be completely closed so that vocalizations are impossible [e.g., (14)], (3) in a published case of laryngospasm (16) where “inspiratory effort became increasingly prominent, accompanied by prominent tracheal movements and inspiratory stridor, also evidenced by increasing EMG artifact in EEG and EKG channels” (no mention of stertor), (4) in obstructive sleep apnea where there is no mention of stertor (60), and even (5) in breath holding divers where no sounds are reported (61). Vilella et al. conclude “breathing related rhythmic muscle artifact is more indicative of breathing effort than obstructed breathing and thus may not be a particularly useful biomarker for SUDEP,” but this is exactly the point—such effort would occur during obstructive and not central apnea. In our opinion, the arguments supporting obstructive apnea as the link between seizure activity and events leading to SUDEP are strong enough to warrant the first aid recommendations presented here.

## Airway Management

Most healthcare facilities have emergency airway protocols and even difficult airway rapid response teams (62–64) to deal with non-routine airway management. Emergency airway management, from repositioning, to relieving an obstruction, to bag-valve mask ventilation (BVM), rapid sequence intubation (RSI), and if necessary, establishing a surgical airway are all part of the armamentarium of clinicians skilled in airway management [e.g., (65, 66)]. Sedative hypnotics and paralytics can often aid in positive pressure mask ventilation. Depending on the situation, these agents are often used in the controlled environment of rapid sequence intubation [e.g., (66, 67)]. The absence of intravenous access and the immediate unavailability of these medications compound the challenges of dealing with a difficult airway (68, 69). Reestablishing ventilation and the success of a return of spontaneous ventilation depends critically on the time spent without oxygen as cardiac failure, and long-term neurologic deficits can result as possible consequences even after resuscitation. Aggressive airway management is key, and anticipation of airway difficulty can save critical minutes.

The vast majority of SUDEP cases occur outside a hospital where airway management is even more challenging. One option, not intended to substitute for the steps in an emergency airway protocol, but rather as a tool in the absence of hospital resources, is the laryngospasm notch maneuver (70). Often used by anesthesiologists to relieve laryngospasm after extubation and emergency medicine physicians, the laryngospasm notch maneuver consists of applying strong pressure behind the angles of the jaw in a supine individual together with a forward jaw thrust (71). It is described by some as universally successful in breaking laryngospasm. As reported by Larson, the middle finger of each hand is placed in the notch below the pinna of the ear between the mandible and the mastoid process of the temporal bone. Very firm pressure toward the base of the skull combined with forward pressure on the jaw (the jaw thrust) combine to abolish laryngospasm (70). The maneuver, in addition to shifting epiglottic structures for a better airway (71), is a painful stimulus that may relax the vocal folds (70). The laryngospasm notch maneuver requires no equipment, may break the laryngospasm early, and is simple to learn and apply (e.g., https://www.youtube.com/watch?v=eIdWRYOQenQ).

## Proposed Resuscitation Guidance

Based on the recent recognition of obstructive apnea as the proximal cause of death during seizures, we propose three additions to the standard first aid algorithm for seizures. In contrast to the age-old emergency response teaching for patients having a generalized tonic clonic seizure—allow the seizure to terminate on its own prior to any airway-related interventions—we posit that the life-threatening consequences of obstructive apnea warrant immediate preventative and interventional measures during and after a seizure. At the same time, none of the proposed additions require manipulating the oral cavity of a patient having a generalized seizure, in keeping with the traditional rationale to protect both patient and responder from injury (18–21).

**Figure 1:**
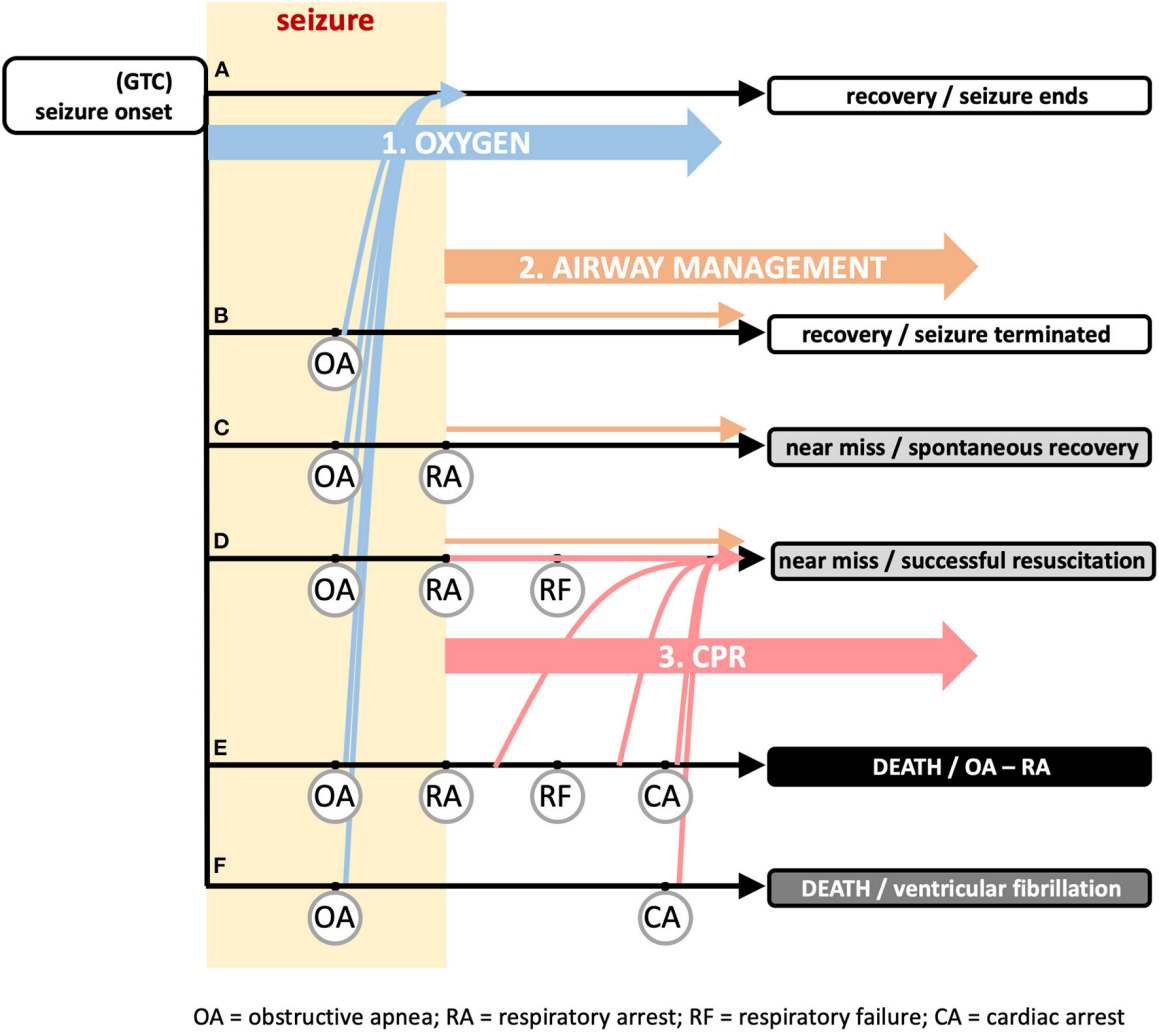
Recommended additional first aid guidance and timing to reduce sudden death risk associated with seizure activity. The vast majority of seizures end spontaneously (track a) with a rapid return of autonomic, cardiac, and respiratory function to baseline levels. The general first aid guidance of “(1) STAY with the person and start timing the seizure, (2) keep the person SAFE, and (3) turn the person onto their SIDE if they are not awake and aware” (https://www.epilepsy.com/learn/seizure-first-aid-and-safety/first-aid-seizures-stay-safe-side) is sufficient for these cases. Onset of generalized tonic clonic (GTC) seizure (left) can be followed by a succession of physiological “landmarks” illustrated in tracks b–f that are defined by the set of landmarks included in the track. The onset of obstructive apnea (OA) can occur suddenly and unexpectedly due to laryngospasm and is shown occurring on all (tracks b–f). OA can persist until the point of respiratory arrest (RA), defined as the time at which attempts to inspire stop. RA is shown in (tracks c–e). If the seizure does not end on its own, hypoxemia from OA can cause the seizure to terminate (yellow rectangle denotes duration of seizure, ending by either mechanism). The hypoxemia developed from OA can be severe enough that the point of respiratory failure (RF) is reached. Within the period between RA and RF, a spontaneous return of respiration is possible if an airway becomes available; after the point of RF, the spontaneous return of respiration is not possible, even if an airway becomes available; resuscitation by a care giver is essential. Continued hypoxemia will lead to cardiac arrest (CA) and death (track e). In track f, the global hypoxemia triggers ventricular fibrillation and death by cardiac arrest. Early oxygen (intervention 1; blue arrows), i.e., applied during the period between seizure onset and the onset of OA, will not prevent OA, but will shift all tracks b–f to track a where the seizure ends on its own and the OA abates as the seizure abates. This is illustrated by the blue arrows from tracks b–f jumping to track a without eliminating OA. Airway management (intervention 2; orange arrows) will ensure one of the recovery or near miss outcomes (tracks b–d), depending upon when the airway opens on its own or if the airway is actively manipulated. CPR (intervention 3; red arrows) is useful even without active airway management, but depends on an airway (spontaneously open or managed). As documented in the literature, this can be applied within several minutes after RA to successfully resuscitate an individual. In track f, the airway is important, but not the critical issue for resuscitation. Ventricular fibrillation will rapidly terminate the seizure and thus release the airway, but necessitates defibrillation for resuscitation. Each intervention has its own separate impact or can be combined. Adapted from Stewart et al. (17).

The three proposed additions are illustrated in Figure 1, together with potential outcomes following a generalized seizure with mechanistic landmarks of obstructive apnea (OA), respiratory arrest (RA), respiratory failure (RF), and cardiac arrest (CA) (adapted from (17):

1. Early oxygen protects against prolonged obstructive apnea and limits desaturation. In a rat model, access to an atmosphere enriched with oxygen approximately doubled the time between the onset of obstructive apnea and respiratory arrest (56). By delaying the time to respiratory arrest, oxygen exposure prior to the onset of obstructive apnea dramatically increases the probability that the seizure ends spontaneously and safely. As desaturation is a major factor in all SUDEP mechanisms (17), the prophylactic benefit of oxygen is compelling. A number of epilepsy monitoring units apply oxygen routinely at the first signs of a seizure.
2. Aggressive airway management may become suddenly and unexpectedly necessary during or after a seizure. The emergency first aid protocols for airway management are excellent, but we believe that emergency medical personnel and care givers must be made more aware that airway status can rapidly change without evidence of positional issues or obvious aspiration danger. Obstructive apnea due to laryngospasm will be accompanied by vigorous attempts to inspire, which can look like clonic movements or successful breaths, but the force of laryngospasm will diminish as the seizure abates and will eventually permit lung inflation by resuscitative effort. The time at which evidence indicates laryngospasm abates is between the point at which the seizure ends and the point of respiratory arrest. Lung inflation may not be efficacious until the laryngospasm has fully abated closer to the point of respiratory arrest. We are not suggesting that large numbers of seizure patients will need intubation. We are emphasizing that (a) an understanding of what to expect will inform decisions about intubation and, more importantly, that (b) the laryngospasm will abate or can be broken with the laryngospasm notch maneuver to permit ventilation in cases where obstructive apnea is suspected.
3. CPR alone, in or out of the hospital, can be sufficient. As discussed above, CPR was effective in all cases reported in the MORTEMUS study when applied within 3 min of respiratory arrest (10). Such resuscitation would only be possible if the airway had spontaneously opened, reinforcing the point that the airway will open when the seizure ends and the stimulus for laryngospasm ends. Chest compressions are important because, as described earlier, cardiac contractility near the time of respiratory arrest is severely impaired. CPR should be continued until the individual demonstrates a return of spontaneous circulation, and ventilation assistance should continue until the individual is clearly breathing on their own.

## Limitations

A number of critical research questions remain, including how to help individuals at risk for SUDEP and their caregivers anticipate life-threatening events, and approaches to resuscitation that can be utilized anywhere. A greater understanding of the mechanisms by which seizures cause laryngospasm sufficient to completely obstruct the airway will also enable potential preventative strategies. One could argue, however, each two points: (1) that the vast majority of SUDEP cases are unwitnessed, and thus no first aid can be attempted, and (2) that the vast majority of witnessed seizures, whether they occur in the hospital or out of the hospital, are not fatal and thus do not require a change in first aid recommendations. Neither of these arguments, in our opinion, are arguments against simple, safe recommendations (including increasing awareness) that can prevent death in the small number of cases whose lives depend on modified first aid when it is available.

At this point in time, the best prevention is adequate seizure control (11), but we believe early oxygen can help reduce a number of other potential consequences. For example, seizure-associated ventricular fibrillation (VF), which is a less common cause of SUDEP, can be triggered by global cardiac hypoxia (17, 72, 73). Additionally, it has long been thought that in patients with epilepsy, sudden cardiac arrest usually occurs in relationship to an acute seizure (9, 74, 75). Although epilepsy is a risk factor for sudden cardiac arrest (76–78), Stecker et al. (77) found that the majority of patients with epilepsy who sustained a sudden cardiac arrest, did not have a seizure just prior to their arrest. In the Oregon Sudden Unexpected Death Study, only about 1/3 of patients with history of epilepsy and a witnessed arrest had evidence of seizure activity before the arrest (11/32) (77). The epilepsy patient population also had >4-fold poorer rate of survival to hospital discharge after attempted resuscitation (77). Hypoxia from laryngospasm for example, can both precipitate ventricular fibrillation and negatively impact outcomes even after resuscitation.

## Conclusions

We believe that research on SUDEP has converged to permit an understanding of why early intervention with CPR has been successful in epilepsy patients and that vigilance with regard to assessing airflow and inspiratory effort can save lives, in spite of the sudden, unexpected occurrence of life-threatening airway obstruction.

## Author Contributions

All authors listed have made a substantial, direct and intellectual contribution to the work, and approved it for publication.

## Conflict of Interest

The authors declare that the research was conducted in the absence of any commercial or financial relationships that could be construed as a potential conflict of interest.
